# The experiences and strategies of parents’ of adults with anorexia nervosa or bulimia nervosa: a qualitative study

**DOI:** 10.1186/s12888-021-03345-5

**Published:** 2021-07-07

**Authors:** Jannike Karlstad, Cathrine Fredriksen Moe, Mari Wattum, Berit Støre Brinchmann

**Affiliations:** 1 Faculty of Nursing and Health Sciences, Nord University, Bodø, Norway; 2 Nordland Hospital Trust, Bodø, Norway; 3 KUN Centre for equality and diversity, Nordfold, Norway

**Keywords:** Anorexia nervosa, Bulimia nervosa, Parents, Caregivers, Health services, Qualitative research, Grounded theory

## Abstract

**Background:**

Caring for an individual with an eating disorder involves guilt, distress and many extra burdens and unmet needs. This qualitative study explored the experiences of parents with adult daughters suffering from anorexia nervosa or bulimia nervosa and the strategies they adopted. A subsidiary aim of the study was to explore the relationship between the caregivers’ perceived need for professional support and the support they reported receiving in practice from the health services.

**Methods:**

Semi-structured interviews were conducted with 11 mothers and fathers from across Norway. Data collection, coding and analysis was conducted using the principles of constructivist grounded theory in an iterative process. The main concern shared by participants was identified by this process and their “solution” to the main concern then formed the content of the core category.

**Results:**

″Wearing all the hats″ emerged as the core category, indicating that the parents have to fulfil several roles to compensate the lack of help from health services. The three subcategories: “adapting to the illness”, “struggling for understanding and help” and “continuing to stay strong” described how the participants handled their situation as parents of adult daughters with eating disorders.

**Conclusions:**

In daily life, the parents of adults with eating disorders have to attend to a wide range of caregiver tasks to help their ill daughters. This study suggests that the health services that treat adults with eating disorders should be coordinated, with a professional carer in charge. The parents need easy access to information about the illness and its treatment. They also need professional support for themselves in a demanding situation.

## Background

It has been found that families caring for an individual with an eating disorder bear a heavy burden and are subject to severe psychological stress. Parents experienced guilt, distress and unmet needs, as well as being shut out of the patient’s treatment [1–3]. Families of individuals with eating disorders are reported to have worse family functioning than control families, though little evidence has been found for a typical pattern of dysfunction [4]. It has also been found that families of individuals with eating disorders share some of the same concerns and needs as families of individuals with schizophrenia, including problems communicating with the ill individual and worries about the future caused by the course of an illness that is often chronic in nature [5, 6]. Despite the caregiving burden being reported as heavier among those caring for individuals with eating disorders than it is among those caring for individuals with depression or schizophrenia, less support was provided for eating disorders [5–7].

The need most frequently reported by those caring for individuals with eating disorders is support and counselling by a professional [1–3, 5–7]. Interventions, such as practical and tailored information from mental health professionals and family-based treatment and skills training for family members, have led to moderate reductions in emotional distress and the burden on carers [8–10]. Psychoeducation for caregivers was evaluated as possible means of reinforcing their ability to support the ill family member [11]. Participants in multi-family therapy for adults with eating disorders reported positive experiences from contact with other families in similar situations, as they were able to discuss and regulate difficult emotions [12, 13]. Involvement of family in the treatment of eating disorders is most common in the case of children and adolescents. However, the involvement of family members in the treatment of adults also led to positive outcomes [9, 14]. It has been argued that irrespective of the stage of the illness, the impact of the eating disorder on the caregivers should determine the type of interventions needed [8, 10, 14].

When children were transferred from paediatric to adult care, the parents experienced conflicting demands and insecurity in their parenting role. Parents experienced greater responsibility for providing care when their child reached adulthood, as the focus changed from family-orientation with frequent follow-ups, to individual focus with fewer follow-ups in adult psychiatry [15, 16]. Both young adults with eating disorders and their parents found this transfer distressing and confusing, as the service providers’ expectations of family involvement and personal responsibility were inconsistent [17]. It has been reported that individuals with eating disorders find independence and adult responsibility difficult, hence they often live with their primary family longer than their peers [18–20]. An association has been found between the duration of the illness and the caregiving experience, as the caregivers had to adapt to the illness to a greater degree when it persisted longer [1, 2]. It has also been reported that hopes of recovery and external assistance tend to be higher during the early phases of the illness [2, 18, 21, 22].

Those caring for adults with mental illnesses have found the information given by the health services to be inadequate and in short supply. Information was held back, even though the patient had consented to the family being informed. A lack of cooperation between the health services and the family, as well as between mental healthcare professionals, was also reported [23, 24]. Everyday life for families living with a young adult suffering from mental illness was described as involving a balance between support, care, work commitments and having a social life [20, 24]. Parents of adults with eating disorders were highly involved in caring, but received minimal practical support themselves [20, 25]. Some reported being left alone with the caring responsibility [26]. Their needs for information about the eating disorder and support from a network or organisation, were reportedly not met [27–30].

The current study builds on findings from a study, carried out in 2020 by the authors in this project group, on parents’ experiences of having an adult daughter with an eating disorder [26]. Eleven parents were interviewed and the main finding was that they felt isolated in their lives, as everything revolved around adjusting to the daughter’s eating disorder. It was also reported that the help from the health services was insufficient and the parents felt excluded [26]. This study explores the experiences of the parents of adult daughters with an eating disorder in further depth and looks more closely at their experiences with the health services. A guideline written by the Norwegian Directorate of Health [31], emphasises that adult relatives are important contributors to the treatment of ill family members. Relatives of individuals with life-threatening or chronic illnesses, such as a severe psychiatric diagnosis, are defined as relatives with special needs [31]. A gap has been identified between relatives’ need for support and what they actually received from the mental health services. Relatives reported not being involved in treatment, advice or decision-making regarding their family member [24, 32, 33].

The aim of this study was to add to the body of knowledge about how parents of adults with eating disorders experienced their situation in everyday life. What strategies did they use and what were their experiences of the health services? A further purpose was to increase knowledge of the relationship between caregivers’ needs for professional support and the support they reported receiving, in practice, from the health services.

## Methods

### Design

This qualitative study used the principles of constructivist grounded theory [34] to gather and analyse data to explore the experiences and strategies of parents of adults with eating disorders. The aim of the study was to explore how the participants handled their daily life situation as parents of adult daughters with eating disorders. A grounded theory approach was chosen because it is considered to be an appropriate tool for exploring actions and interactions in particular settings, when the aim is to explain rather than describe [35]. The avoidance of any pre-formulated hypothesis in grounded theory means that the explanation is generated from the data; the analyses is ‘grounded’ in the data. The simultaneous collection and analysis of data helped the authors to focus on developing concepts about the data. The analyses also helped to shape the next stage of data collection [34, 35].

The data collection methodology should be based on the research question, as well as the accessibility of data [34]. Individual semi-structured interviews [36] were considered to be appropriate tools for gathering the data for this study. Active use was made throughout the research process of memos, reflections on the research process and on codes appearing in data. Ideas for an initial analysis emerged from these deliberations. Following the principles of grounded theory, an iterative process of data collection, coding and analyses was employed. The main concern shared by participants was identified and their “solution” to the main concern then formed the content of the core category [34].

In constructivist grounded theory it is acknowledged that what one discovers in data is partly driven by one’s own perspective [34]. This version of grounded theory differs from the original grounded theory which emphasized that the researcher should have an objective approach to data [37, 38]. According to Charmaz [34], the objects of study include the researchers themselves. Theories are constructed through researchers’ involvement and interaction with the participants, as part of the overall research practice. The constructivist approach offers an interpretation, not an exact picture, of what is studied [34].

### Co-researchers

Two co-researchers (MW, RAS) were involved as consultants in the project. One has personal experience of an eating disorder; the other has experienced being the mother of a daughter with an eating disorder. The co-researchers were involved in developing the research questions and the interview guide. They also participated in discussing and planning the project as a whole. MW participated in the final analysis of the results. Their ability to view the results from a different angle, based on their experience, was a valuable contribution throughout the research process.

### Participants

Participants were mothers and fathers of adult women (over the age of 18) with anorexia or bulimia. In the majority of cases, onset of the illness occurred in their early teens, though a few were in their early twenties. Long duration (± 10 years) of illness was common for the group. A couple of the participants described their daughters as now having control over their eating disorder. The majority of the participants described their daughters as fluctuating between better and worse periods in their eating disorders. Some of the daughters were currently under treatment.

Participants were recruited by one inpatient and three outpatient eating-disorder and general psychiatrics units, as well as two organisations providing information and support to patients and families on eating disorders. The units and organisations conveyed information about the project to prospective participants, who voluntarily signed up to participate. Consent for participation had first to be given by the daughter affected by an eating disorder. Participants were recruited from different counties within Norway. Eleven individual interviews were conducted, with parents from seven families. Table 1 describes characteristics of the participants.

**Table 1:** Characteristics of participants

Participant (age 52–70)	Marital status	Number of children	Daughter (age 24–32)	Diagnosis of daughter	Daughters’ current living situation
Mother 1 Father 1	Married	2	Daughter 1	AN	Not living with parents
Mother 2	Married	2	Daughter 2	AN	Not living with parents
Mother 3	Divorced	1	Daughter 3	AN	Living with parents
Mother 4	Divorced	2	Daughter 4	AN	Partly living with parents
Mother 5 Father 5	Married	2	Daughter 5	AN	Not living with parents
Mother 6 Father 6	Married	4	Daughter 6	BN	Not living with parents
Mother 7 Father 7	Divorced	3	Daughter 7	AN	Not living with parents

*Note: AN* Anorexia nervosa, *BN* Bulimia nervosa

### Data collection

Semi-structured interviews were conducted by asking a few interview questions, so that participants had the opportunity to steer the direction of the interview [34, 36]. An interview guide was used, with a few predetermined open-ended questions. The questions were as follows:

- Can you describe your experiences as the mother/father of an adult daughter with an eating disorder?
- How is your family made up and what is your daughter’s ED diagnosis?
- How does/did living close to a family member with this illness affect you and your family?
- Are there/have there been any main challenges in your family?
- Are you using/have you used any particular strategies for handling your situation as the mother/father of a daughter with an eating disorder?
- Have you had any experience of the health services in this context? If so, please describe your experiences?

The guide was adjusted as new themes were brought up [34]. During interviews, participants were asked about their coping strategies and experience of the health services. Repeated themes from the first interviews included “not being taken seriously by the health services” and “receiving help was based on luck”. More pointed questions were then developed by following up on codes from the early interviews [39]. One question constructed after the first interviews in order to refine participants’ precise needs was: “What specific help would you like the health services to provide?”

Seven interviews were conducted face-to-face, the remainder by telephone (due to Covid-19). All interviews were audio recorded and transcribed verbatim. They each lasted between 25 and 150 min. The majority lasted for about 1 h. Interviews and transcriptions were conducted by first author. The quotes from the participants used in this article have been translated into English from the transcriptions of the interviews in Norwegian.

### Data analysis

The data were analysed using principles of constructivist grounded theory [34]. First, the transcriptions were initial coded, sticking closely to the data by studying words, lines and segments. In vivo codes i.e. participants’ own terms, words or sentences were used where appropriate. The next stage of analysis was focused coding, where the initial codes were studied and compared. The focus was on the message from the initial codes and the comparison between them. The focused codes were constructed based on the frequency of the initial codes or their significance for the aim of the study. In order to facilitate subsequent categorisation of the data, decisions needed to be made about which initial codes made the most analytical sense. The voice of the researcher becomes more evident in this phase, as the focused codes are based not only on the participants’ statements, but on their relevance to the topic as well. As Charmaz & Thornberg put it: “Define the most important codes as focused codes” [39 p4]. The focused codes were then compared and merged into preliminary categories. These categories consisted of groups of focused codes with similar characteristics [34].

Several memos were written about the development of the categories, addressing issues such as: “What is the most essential element in the data?” and “What do the categories consist of?”. The theme “not getting adequate help” was very apparent after the first interviews: “Does this mean that this help is unavailable, or does the help the parents need simply not exist?”. The message from the emerging categories was still unclear, so they needed to be elaborated and refined and more interviews were conducted. After further data collection a main category was identified, with three related subcategories. The final interviews added some additional details to the categories, though not any significant new information. Most of the interviews were lengthy and they yielded rich data, which provided in-depth insights into the phenomenon that was being explored.

JK conducted the coding process. After continuous, iterative comparison of data, codes and preliminary categories a core category was developed. To ensure consistency, the development of the core category was a collaborative process involving the authors JK, CFM, MW and BSB.

In constructivist grounded theory it is acknowledged that what one discovers in data is partly influenced by one’s own perspective [34]. Reflexivity is required throughout the research process. This is exercised by explicating assumptions that are taken for granted and then being conscious of how hidden beliefs can enter the process. The researcher must strive not to see one perspective as the only possible interpretation [39]. The first author has several years of experience in clinical work with patients with eating disorders in an inpatient unit. In practice, reflexivity was exercised through the authors and co-researchers discussing and reflecting on the data together, using the first author’s memos as the basis.

### Ethical considerations

This project was approved by the Regional Committees for Medical and Health Research Ethics, REK Vest, Norway (2019/762) and by the Norwegian Centre for Research Data, Norway (2020/222970). Participants gave informed written consent to participate voluntarily in the project and had the opportunity to withdraw at any time. At the Ethical Committee’s request, the first consent required was from the daughter affected by eating disorder. Participants’ confidentiality was protected by anonymising the data. Information about their identities was stored securely in accordance with data legislation and university procedures.

## Results

*Managing the role of parent without adequate help* emerged as the parents’ main concern. This theme had already started to become apparent from the very first interviews. To a greater or lesser degree, all the parents talked about the lack of adequate help from the health services. Some said they had hardly received any help at all. Others had received some help, but it was not sufficient and often not when they needed it.

### “Wearing all the hats”

To manage this main concern, the core category *“wearing all the hats”* emerged as a metaphor for the parents needing to fulfil multiple roles to compensate for the lack of help. In their situation, having to wear many hats means having several different roles or tasks to perform: “*In addition to my work … I am the one who has to be alert to her losing weight. It is my responsibility to see that she takes her medicine. I have to make sure she does everything she is supposed to …*” *(M6). “Wearing all the hats”* is an in-vivo code from the last interview, where one of the mothers explained how she was taking responsibility for coordinating health services for her daughter, as well as having to play the role of mother, nurse, doctor and psychologist. The parents experienced overload and insecurity in fulfilling these roles: “*It should have been taken off our shoulders. We are not health care workers. We are just relatives. Yet in a way we become health care workers too” (M6).*

Three related subcategories also emerged: *adapting to the illness*, *struggling for understanding and help* and *continuing to stay strong.* These phrases describe the main content of the multiple roles played by the parents as they managed their main concern during the successive phases of the daughter’s illness. All the participants talked about how their daughters’ illnesses began. In most cases the illness started in the teenage years and then continued into adulthood, by which time many of the daughters were no longer living at home.

### Adapting to the illness

When the parents realised that their daughter was struggling with an eating disorder they started adapting to the illness and began monitoring the daughter’s physical health, helping her with food and meals and reducing social contact with family and friends as dictated by the daughters’ needs. The hats they wore were those of ‘nurse’, ‘dietician’ and ‘guard’.

*“I felt I had to take on so many responsibilities that were far beyond being a mum … and as she is your own daughter you do not have a professional approach” (M7).* The parents found that adapting to their daughter’s illness meant taking on a great deal of responsibility. Some of the roles they had to take on placed heavy demands on them, as they were now entrusted with a responsibility they had not sought. One mother became responsible for her daughter’s meals after she came home from the hospital: *“She brought a dietary list of what to eat and so on. It became my job to make sure that it actually happened and this being supposedly my job really did not work that well” (M3).* Some food and meal routines consumed a great deal of time during the days and these situations caused considerable tension and distress: “*It affects everything, from morning to evening. Dinner, diets, meals …” (F5).* Parents played the roles of dietician and guard, as they monitored their daughter’s meals.

Since a lot of time and resources was spent on the ill daughter, several of the parents felt guilty because they were not paying enough attention to siblings: *‘Then there are siblings, two sisters, but most of my attention is on following up on the ill one’ (F7).* Several of the parents had to forego their social life outside the family. Participating in unplanned activities and travelling became difficult. Their roles as carers became more prominent than their roles as adult, partner or friend: “*Everyone else has older kids who manage to take care of themselves, … who else brings along their 19-year-old when going away on a weekend with friends?” (M2).* “*… I had found a new partner, but it was difficult for him that she took up so much space, so the relationship ended” (M7).*

Several of the parents were resourceful in terms of network, economy and job flexibility, so they managed to play the various roles demanded of them. *“I have been very lucky as I have had both the economic ability and opportunity to be so close to her …” (M3).* Some arranged for one parent, or both, to stay at home for periods. Openness within the family was also described as a resource: “*… and we talk about both unpleasant and pleasant things, so our home is a very open environment. For us that has been a strength” (M5).*

The group of fathers was characterised by being more preoccupied with practical matters and making things happen, than the group of mothers. The mothers were perceived as being more emotionally involved in the process.

### Struggling for understanding and help

While arranging appropriate help for their daughter and support for themselves, the parents also needed to gain better personal understanding of the eating disorder. While acting as carers in the home, the parents were also searching for professional help from outside. To make this happen, they had to don the hats of ‘struggler’ and ‘coordinator’.

A father started to feel insecure, while caring for his daughter on his own: “*She became weaker and I stood alone without much help from anyone. This (illness) is unknown to most of us when it occurs. You do not have anything to compare it with. You have no experience or references. You feel helpless, do not know what to do or whether you are pushing the right buttons or making the right moves” (F7).* When the daughters received help from the health services, several of the parents mentioned challenges concerning confidentiality: *“… she is of legal age, so they should not refer to us (F1).* Experiencing that the health services refused to inform them left the parents uncertain: *“... When should we call for urgent help? When should we do all these things?” (M3).* The issue of having the responsibility for her daughter, while not being involved or informed by the health services, was addressed by this mother: *“Then I think that if we as parents are going to have that responsibility and there is supposed to be user involvement, we actually need to know a little about what to do” (M3).*

One mother was astounded when she started to understand the severity of the illness, but was not taken seriously by health care services: *“… I did realise how ill she was, but they just dismissed me completely ... I wish that they had taken it seriously right away. … one is sent back and forth. No one talks to each other and no one has the authority to …” (M2).* A father’s fight for help for his daughter became an additional burden in their situation: *“I have experienced parts of the health care services as a fierce opponent during some periods … That we somehow had been involved …” (F1).* Two mothers also described the help-seeking process as a struggle: *“Whereas with this problem … you knock on every door, but you are rejected” (M7). “It has always been a struggle getting help … I tried to persuade them to refer her to a place where she could receive help. … I felt that I was not being taken seriously” (M3).* The parents assumed the role of fighter. They had to act in a determined way and some had to raise their voices in order to be heard and obtain help: “*If they feared I would become angry, then they started offering conversations. I have the impression that I am only listened to if I raise my voice and become strident” (M3).*

A father was satisfied with the help the daughter and the whole family received from the hospital, but was left with many unanswered questions: *“… so I am left with a lot of questions. I want confirmation … whether it is right or wrong” (F5).* Some of the parents were satisfied with parts of the help received. Instances included admission to different institutions and multi-family therapy. Some suggested that conversation groups for parents would be helpful and those who participated in societies for eating disorders found that valuable. Parents who found themselves in the coordinator role, wanted better official coordination by the health services. A majority of the parents experienced that their daughters fluctuated between better and worse periods in their illness. Achieving full recovery seemed to be a daunting task. They requested longer term and more accessible treatment for adults with eating disorders. *“… could she not have received psychological treatment again, to help her to move on and support herself into the future as well? (F7).*

### Continuing to stay strong

The parents managed to play multiple roles to a greater extent during the first phase of the illness. Many of them described feeling more optimistic about the prospects of recovery during the early stages and this led them to act more proactively. Then the years went by and the majority of the daughters were not fully recovered. Adaptation and struggling shifted into coping with a long-term situation and the parents now wore the hats of ‘survivor’ and ‘overcomer’. The parents were becoming weary, but had to stay strong to cope with the continuing situation of the daughter’s illness. Even though a majority of the daughters had moved out from home, the caregiving responsibility and worrying continued for most of the parents. Some of the parents sought help for themselves, which they needed in order to continue to live with the situation. Several of the parents continued working, as work provided them with a break and gave them an outside focus in everyday life. Some needed sick leave from work to cope with the situation.

The illness was often of long duration and this had a huge impact on the families’ lives: *“We kept on going for seven years. You have to have some life of your own as well. It is very tiring. … having to live in accordance with the situation” (F5).* The parents realised that it takes time to recover from an eating disorder and some sufferers may never recover. Some parents had mental breakdowns. Some became physically ill, but somehow managed to live with the situation. After years had passed, one mother finally sought help for herself: *“I am not the way I usually am. The scary part is that when other people somehow expect me to be sad, to cry or laugh … the psychologist said that as you have been exposed to many shocks, been on standby for so long, the body just shuts down because it thinks there is another shock coming” (M2).*

Some had come to realise that their daughter would never recover completely. Years of worry take their toll: *“I feel as though I have something inside of me that is so heavy” (M5).* After several years, these parents settled down and learnt to live with the situation: *“I have hung in there, not collapsed … do the best you can, do not think so much about worst-case scenarios and things you have no control over” (F7). “It is probably not something we can escape either. We have probably been assigned to this … , so just have to hang in there I suppose” (M6).*

Openness with one another also helped family members to stay strong. While some of the families had always had an open atmosphere, others found that the eating disorder contributed to this: *“What it (the eating disorder) may have done for us is that there is nothing we have not talked about. The four of us are very closely connected, because we have been through so many sad and difficult things” (M2).* Those who managed to keep an open dialogue at home and could also be open about their situation at work found this a relief: *“I have chosen to be open at work, otherwise people would not know why I leave a meeting” (M2).*

When looking back at all the years the illness had lasted, the parents felt that resourcefulness was crucial. Without it, they would have been unable to help their daughters in the ways they did. The parents stated: *“If we had not been resourceful and had money, I do not think I would have had my daughter today” (M2).* Several of the parents continued to help their daughters financially: “*Does all that she manages to … , but she becomes financially dependent on us anyway, we help her with pleasure” (F1).*

### Summary

The parents’ focus in daily life was on the many caregiving tasks required to help their ill daughters; They had to wear all the hats. They felt that they had become substitutes for the health service carers by taking over their responsibilities. The parents spent time and effort figuring out where and how to seek help for their daughters and had to take on the task of coordinating the available services. Even if the ill daughter moved out from the family home, many of the caregiver responsibilities continued for the parents. Being resourceful and maintaining openness within the family helped the parents to cope with the long-lasting illness. The parents’ roles changed gradually over time, but their role as carer was always prominent.

## Discussion

### “Wearing all the hats”

The current study found that the participants were required to fulfil multiple roles. They had to “wear all the hats″ to compensate for the lack of adequate help from the health services for their daughters with an eating disorder. The parents were required to play multiple roles at the same time. According to Merton’s role theory [40] each social status involves not a single role but several roles - a role-set. Individuals can experience role strain if too much is required of a certain role. When one or several roles are contradictory, the individual possessing the role(s) can experience role conflict. At the same time as the parents in the current study were taking care of their ill daughters by monitoring their psychical health, facilitating meals and searching for professional help, they had other concerns. They had to care for their other children and family members, hold down a job and maintain friendships and social life. Fulfilling multiple roles, or wearing several hats, often leads to overload for the individual concerned [40]. The parents experienced role conflict in handling their everyday life with a daughter suffering from an eating disorder.

An individual subject to role conflict finds that different roles require different and sometimes contradictory behaviours, or changes in the situation require changes in behaviour [40]. The role-sets of the parents in the study were to some extent determined by the phase of the illness. During the first years of the daughter’s illness the parents wore the hats of ‘carer’, ‘nurse’, ‘coordinator’ and ‘struggler’, while in the later phases the roles were more about coping with a long-term situation wearing the hats of ‘survivor’ and ‘overcomer’. However, the ‘carer’ hat was always on regardless of phase. The balance between support, care and help, alongside managing work and enjoying a social life, is discussed in the study by Aass et al. [24] on families of individuals with mental illnesses. Fox and Whittlesea [20] reported similar findings in their study of caregivers and the ways in which they accommodate the wishes and needs of anorexia sufferers. Here it was found that the caregivers had to fulfil numerous perceived obligations at the same time.

The current study found that the mothers were perceived as being more emotionally involved in the caregiving process, while fathers were more preoccupied with practical matters. These differences between the roles of mother and father have been reflected in previous studies [2–4].

### Contradictory expectations

Certain expectations or norms accompany each position or set of roles filled by the individual [40]. The norms that apply when wearing the hats of ‘parent’, ‘health carer’, ‘fighter’, ‘partner’, ‘worker’ and ‘friend’ involves differing and contradictory expectations [40]. Wearing the ‘parent’ hat was described as challenging because most of the participants had more than one child to take care of. Given the demands on the parents, it was difficult to give the siblings the care and attention required. Previous research has shown that healthy siblings of individuals with eating disorders reported a lack of care and space for them in the family [20, 41, 42]. The parents in the current study described feeling guilty because they were unable to follow the norms when wearing the ‘parent’ hat’. Another area where norms were difficult to follow was associated with the parents’ roles as partner or spouse, which could be challenging as most of their attention was directed towards the ill daughter.

Parents generally found wearing all the hats demanding. They also felt insecure in some of the tasks they were performing. They were uncertain what was expected of them in the new roles they had to fulfil. They lacked the expertise of a professional health carer and they missed having confirmation of the rightness or wrongness of their actions when caring for their daughters. Previous research has shown that anxiety often becomes the governing factor in families with a family member suffering from an eating disorder. This can lead to limited interactions outside the family [1, 2], as the parents in the current study described in the context of social life. Some of the parents took sick leave from work; others wanted to spend more time at work even if it stretched their capacity. Several experienced that being husband and wife and living their full life as adults was taken away from them. Holiday travel became difficult or impossible. Previous research has described similar challenges for families of individuals with an eating disorder, with restricted social life and little capacity to respond to their own needs [2, 3, 8, 9, 20, 27, 43].

Honey & Halse [44] examined the strategies that parents were using to cope with the situation of having a daughter with anorexia nervosa. The parents of those who had been ill for a long time set boundaries in terms of how much they let the eating disorder affect their lives and they pulled away from the daughter emotionally. Others set boundaries around contact with other people to invest all their energy on their ill daughters. These findings revealed more diversity in the data than in the current study. The parents in the current study mostly set boundaries around their social lives and continued to apply their energy to their daughters. In the Honey & Halse study a larger sample, involving 24 participants, captured a wider diversity. All the Honey & Halse parents were still living with their daughters. Living togehter with the ill daughters probably inflicted more tension and distress on the parents, leading to a greater need to establish emotional separation. By comparison, the majority of the group of parents in the current study were not still living together with their daughters, which made the need to establish distance less urgent.

### Coping with a long-term situation

The parents in the current study managed to wear several hats to a greater extent in the first phases of the illness, when many felt more optimistic about their daughter’s prospects of recovery. Several of the parents then experienced little improvement in the daughter’s illness. Some also lived through relapses. It became harder to follow the norms for each set of roles under these circumstances. The norms for the ‘health carer’ hat became wider and more difficult to follow, because the effort devoted to monitoring their daughter’s health resulted in little improvement over time. The intensity of their efforts to help also diminished, as they found that the professional help their daughters received was lacking or insufficient. The parents adapted their lives to the situation and made the best of it by wearing the hats of ‘survivor’ and ‘overcomer’. Previous research has also found that the duration of the illness has an impact on caregivers and it describes a higher level of hope and quality of life in the beginning [2, 18–20]. Statistics have shown that 20–30% of individuals suffering from an eating disorder develop a chronic condition and the somatic complications that often follow can be long-lasting [45, 46]. Both the current study and previous research have shown that parents often continue to be involved in caring for their children with eating disorders into adulthood [20, 25–27, 29].

The parents in Fox and Whittlesea’s study [20] described the process of caring for their adult daughters with anorexia nervosa as a cycle. They struggled to understand the illness and took on a caring role for their daughter. This often led to direct interventions, conflicts and exhaustion on the part of the parents. A less confrontational approach to accommodating the illness and reducing conflicts was then attempted, resulting in more anorexic behaviour. The parents in the current study described being uncertain about how to help their daughters, but did not say that their adaptation to the illness resulted in more disordered eating behaviour. However, they did not experience any marked improvement in their daughter’s health. In the Fox and Whittlesea study [20] the parents ended up in a cycle, vacillating between accommodation and confrontation. This is similar to the situation experienced by the parents in the current study, as they described finding themselves in an inescapable situation. For both these groups of parents, the responsibility for their ill daughters continued for a long time, often at the expense of their own physical or mental health. Even if their daughter’s health was improving, the parents were always prepared. The carers were always ″on standby″ for stepping back into the caring role [20].

Over time, carers of individuals with eating disorders could start to feel powerless and this diminished their emotional resources [1–3, 20]. Resourcefulness and openness within the family were strengths that enabled the parents in the current study to cope with the long-term situation. In a study on multifamily therapy for adults with eating disorders [47] it was found that open communication improved family members’ understanding of the eating disorder and helped them to understand each other. The individuals with the eating disorders also said that openness helped them to understand their parents’ situation. The parents in the current study described this openness as a resource and a relief.

## Conclusions

The current study shows that the parents of adults with eating disorders had to assume the role of professional caregivers, because their daughters did not receive adequate help from the health services. Wearing all the hats at the same time and having to switch hats frequently, or at times not knowing which hat to wear, became exhausting for the parents. They remained in an extended parenting role when their children reached adulthood, as their adult children did not receive adequate help. These parents are seen as making a significant contribution to the health services. Health services for adults with eating disorders should be coordinated, with a professional carer in charge. Parents of adults with eating disorders should receive as much information as the duty of confidentiality allows about the illness and the treatment that is available. Those caring for individuals with long-lasting eating disorders need professional support for themselves, to help them cope with the situation. Examples are support groups and conversation groups for parents. The participants in the current study relied heavily on having resources such as a good network, a flexible job and adequate finances. Having these resources helped them to care effectively for a family member with a long-lasting eating disorder. Finally, it was concluded that openness within the family is a very important coping factor.

### Strengths and limitations

The sample of 11 participants is small and the findings cannot be generalized for all parents of adults with eating disorders. Nevertheless, the authors experienced a high degree of consistency in the data. The diversity of the group of participants is limited, as the focus is on a specific group of parents of daughters with eating disorders. However, focusing on a small, specific group allows their needs to be clearly highlighted. The alternative of having a more mixed group of participants would have made the knowledge gained more diffuse [3]. The inclusion of parents of daughters with eating disorders is based on prevalence and access to participants. The prevalence of anorexia among females in Europe is 1–4% and bulimia 1–2%, while only 0.3–0.7% males are reported as having an eating disorder [48]. It is highly likely that recruiting parents of sons with eating disorders would generate very few participants. The resulting sample would be too small to compare the parents of daughters and sons. Another potential limitation in the diversity of the group, was that only one of the daughters was reported to have bulimia, while the remainder had anorexia. Only a couple of the daughters in the sample group were currently living at home. The remainder had moved out.

There is always a risk that individuals choosing to participate in research may share certain characteristics and the participants in this study appeared to be particularly resourceful. Because consent for participation had to be given by the daughters affected by the eating disorder, it is possible that these participants do not represent families affected by major conflicts or trauma. It might also be suggested that the requirement for the daughters’ consent limited the sample size of the study. The authors are aware of two families where the parents had intended to participate, but their daughters declined to consent. The same might also have applied to other potential participants. As this study was conducted during the Covid-19 pandemic it is possible that some potential participating families affected by eating disorders were having a particularly difficult time and had little capacity to participate in a research project.

Semi-structured interviews allow participants to share lived experiences as they remember them. The interviews also give them an opportunity to emphasise what is important to them [34, 36]. Interviews conducted by telephone may have differed from those held face-to-face because it is not possible to read body language. Findings reported in this study are in the context of Norway and may therefore not be directly comparable with other cultural settings in certain respects. For example, the healthcare system is country-specific.

Co-researchers’ insights added an extra dimension to the general research methodology and made it possible to view the data that was gathered from different angles. Involving the co-researchers actively throughout the research process strengthened the overall credibility of the study [49, 50].

## Data Availability

The dataset collected and analysed in the current study are not publicly available due to ethical restrictions to maintain the participants’ anonymity. The corresponding author can be contacted on reasonable requests regarding the dataset.

## Abbreviations

AN: Anorexia nervosa
BN: Bulimia nervosa
